# Parental Compliance towards Oral Health Education among Preschoolers with Special Healthcare Needs

**DOI:** 10.3390/ijerph18147323

**Published:** 2021-07-08

**Authors:** Ni Zhou, Hai Ming Wong, Colman Patrick McGrath

**Affiliations:** 1 School of Stomatology, Kunming Medical University, Kunming 650000, China; zhouni@kmmu.edu.cn; 2 Faculty of Dentistry, The University of Hong Kong, Hong Kong, China; mcgrathc@hku.hk

**Keywords:** health education, parental compliance, oral health literacy, pediatric, special needs

## Abstract

Background: Young children with special needs greatly rely on their parents to support their daily living activities; parental compliance may have great impact on the implementation of health promotion activities among those children. This study aimed to investigate the parental compliance towards oral health education (OHE) among children with special healthcare needs (SHCN). Method: The participants were 306 parents whose children had participated in a 24-month OHE program for preschool children with SHCN. The primary outcome of this cross-sectional study was parental compliance towards the OHE program. Parents’ oral health literacy (OHL) was assessed by a validated tool. Parents’ socioeconomic status, attitudes towards OHE topics, perceptions of children’s oral hygiene status, and usefulness of OHE materials were collected via questionnaires. Results: A higher dropout rate was observed among parents who perceived that their children had unfavorable oral hygiene status (*p* = 0.038), or parents who had poor OHL skills (*p* = 0.015). Parental noncompliance was more likely to be observed among parents who perceived that the OHE materials were not useful for their children (OR = 3.63, 95% CI 1.56 to 8.47, *p* = 0.003), or parents whose children had been diagnosed with developmental delays (OR = 5.45, 95% CI 1.59 to18.74, *p* = 0.007). Conclusion: Non-compliance existed among parents whose children had received OHE intervention. Parental compliance towards OHE intervention might be associated with parents’ OHL skills, usefulness of OHE materials, and children’s developmental conditions. OHE programs should be individually tailored to support children whose parents are at a higher risk of noncompliant behaviors.

## 1. Introduction

Children with special healthcare needs (SHCN) typically require additional health services, as they are “at increased risk for a chronic physical, developmental, behavioral, or emotional condition” [1]. Epidemiological studies disclose poor oral health status as well as undesirable oral-health related behaviors to be common among children with SHCN [2,3,4]. Existing evidence also suggests that promoting appropriate oral health-related behaviors among children with SHCN could improve their oral health status, and dentists are recommended to provide oral health education (OHE) to parents of children with SHCN [2,3,5,6].

Health education is an essential component for health promotion activities. The efficacy of health education can be affected by various factors [7]. In a web-based health education project, the researchers elucidated that high parental compliance, i.e., the extent to which the parent’s behavior coincides with the advice of the health worker, can result in desirable outcomes [8]. Meanwhile, participants’ noncompliant behavior can interfere with the efficacy of treatments or interventions, and sometimes, it can even lead to serious consequences [9]. When conducting clinical trials, participants’ noncompliance, such as non-response or loss to follow-up, can reduce the power to detect a true difference between control and test groups [10]. Furthermore, individuals with SHCN are facing a number of challenges in learning new skills, processing complex information, or managing self-care routines, and most of them usually rely on parents or caregivers to support their daily living activities [11,12]. Thereby, parental compliance plays a central role in the implementation of health promotion activities among those children. 

Compliance is considered to be a multifaceted process that is influenced by multiple factors [8,13]. Identifying the predictors of parental compliance can help health workers to be aware of the individuals who might have potential barriers to follow investigator advice. More efficient intervention strategies can be developed accordingly to improve patient adherence and enhance the efficacy of health education programs. Prior studies noted that patients’ noncompliant behaviors could be related to low levels of education, poor socioeconomic strata, poor literacy, or frustration about disease or its treatment [10,13,14]. Travel distance, marital status, and urgency of the surgery could also affect patients’ compliance towards follow-up visits [15]. Moreover, parents of disabled children were likely to show low levels of compliance [16]. However, rare studies have investigated the factors associated with parental compliance towards OHE interventions among young children with SHCN. In this study, parental compliance towards an OHE program was investigated, aiming to identify the potential factors which might be associated with parental compliance towards OHE intervention.

## 2. Materials and Methods

### 2.1. Study Design, Setting, and Participant Recruitment

This cross-sectional study was conducted in Special Child Care Centers (SCCCs) in Hong Kong. The participants were parents whose children had been previously enrolled in a 24-month OHE program, in which preschool children who were diagnosed with multiple disabilities, autism, cerebral palsy, Down’s syndrome, William’s syndrome, epilepsy, a developmental delay, or had other syndromes that required additional healthcare services were recruited. The sampling frame was the list of SCCCs identified from the Government Social Welfare Department in Hong Kong, and 16 SCCCs were selected by using a computerized random sequence generation program. Investigators had illustrated the importance and rationale for cultivating favorable oral health related behaviors to the participants. A package of OHE materials related to toothbrushing training, dietary instruction, and dental visits was delivered to the recruited parents. Children were expected to learn appropriate toothbrushing skills, tooth-friendly eating habits, and the importance and procedures of dental check-ups via reading the materials with their parents at home. The sample size was calculated based on a significant level of 0.05, with a statistic power of 80%. Considering 30% of children might drop out during the 24-month study period, over 300 children were initially recruited [17]. Ethical approval was obtained from the local Institutional Review Board of Ethics (file No: UW 16-012). Written consent forms were signed by parents.

### 2.2. Data Collection

Parental compliance towards the OHE intervention was the primary outcome of this study. It was assessed by two components: (i) participant adherence to follow-ups (whether the participant was lost to follow-up in the 24-month OHE program), and (ii) parental compliance with investigators’ advice (whether parents’ behaviors coincided with the advice of the investigators) [8,9]. Adherence to the follow-up study was observed and recorded by investigators. If parents and their children were absent at the 24-month assessment, it indicated that they did not adhere to the follow-up study. Additionally, in the OHE program, the recruited parents were advised to read the OHE materials with their children at home. Parental compliance with investigators’ advice was reported by parents at the end of the 24-month OHE program via answering the question “Has your child used the health education materials after you received the materials (Yes/No)”. If the participants had never used the OHE materials during the past 24 months, they were indicated to show noncompliance with investigators’ advice. 

Questionnaires were administered to collect information regarding parents’ attitudes towards the OHE intervention, health literacy, perception of their children’s oral hygiene status, and family socioeconomic status (SES). Three OHE topics were involved in the OHE program, namely, toothbrushing, tooth-friendly diet, and preparing children for dental visits in their early childhood. Parental attitudes towards the OHE intervention were rated by answering the following statements: (i) “Tooth brushing can prevent dental diseases”; (ii) “Frequent rewards of sugary snacks can motivate my child to behave better”; and (iii) “Since milk teeth will be replaced by adult teeth, dental treatment in early childhood is not necessary”. Parents were indicated as having negative attitudes towards the OHE topics, if they reported that they “disagree” with the positive statement (statement (i), or “agree” with the negative statements (statement (ii) & (iii)). Additionally, parents were invited to answer the question “How useful do you find the OHE materials” by using a five-level Likert item (“very useful”, “useful”, “neutral”, “useless”, “very useless”). If the materials were rated as “useless” or “very useless”, it indicated that parents perceived that the OHE materials were not useful for promoting health-related behaviors. Parental perception of children’s oral health status was assessed by the following question “How do you rate your child’s oral hygiene status?”. This was rated by a five-level Likert scale (“excellent”, “very good”, “good”, “fair”, “poor”). Children whose oral hygiene status had been rated as “excellent”, “very good”, or good” were assumed to have perceived “favorable” oral hygiene status. Parents’ OHL was assessed by the Hong Kong Oral Health Literacy Assessment Task for Pediatric Dentistry (HKOHLAT-P), which is a validated instrument for examining Chinese OHL in Pediatric Dentistry [18]. HKOHLAT-P draws on a range of literacy and numeracy tasks across three kinds of knowledge (factual, procedural, and conceptual) and cognitive process dimensions (remembering, understanding, and analyzing) to assess caregivers’ oral health knowledge. The total score ranges from 0 to 52, higher scores indicating better functional OHL [18]. Information regarding parents’ occupation status (full-time employed or not), years of education attainment, and levels of monthly income were also collected. 

### 2.3. Statistical Analysis

Statistical analysis was performed by IBM SPSS Statistics 25.0 (IBM Corp., Armonk, NY, USA). Two-sample t test was used to compare the OHL scores between parents with or without noncompliant behaviors. Chi-square test or Fisher’s exact test were performed to assess the differences in the parental compliance among various groups. Parents’ OHL scores, SES, attitudes towards the OHE topics, usefulness of the OHE materials, parents’ perception of children’s oral hygiene status, type of OHE materials, and their children’s primary diagnosis were entered into the full model of binary logistic regression. The independent variable was the presence of parental noncompliant behaviors. By adopting the backward elimination method, odds ratio, and 95% confidence interval were displayed in the final models. All the comparisons were two-sided, and the level of statistical significance was set at *p* < 0.05.

## 3. Results

### 3.1. Characteristics of the Participants

Among the 306 parents, 96 (31.4%) parents were taking care of children with multiple developmental problems, 95 (31.1%) parents had children with autism, 80 (26.1%) had children with a developmental delay, and the remaining parents were caring for children with other conditions that required additional healthcare services. In 107 (35.0%) families, both parents were full-time employees, and 185 (60.5%) families had a household income over HKD 20,000 per month (Table 1). 

Half of the fathers and 123 (40.2%) mothers received tertiary education (14 years or above). Over a quarter (27.1%) of mothers and 61 (19.9%) fathers received no more than nine years’ education. Four (1.3%) parents disagreed with the statement that “Tooth brushing can prevent dental disease”. Nineteen (6.2%) parents agreed that “Since milk teeth will be replaced by adult teeth, dental treatment in early childhood is not necessary”, and 33 (10.8%) parents agreed that “Frequent rewards of sugary snacks can motivate my child to behave better”. A total of 49 (16.0%) parents had showed negative attitudes towards at least one of the involved topics.

Three sets of OHE materials were delivered to the participants, and 301 parents (response rate: 98.4%) had rated the usefulness of the OHE materials. Of those, 68 (22.6%) parents perceived that at least one of the issued OHE materials were not useful. Children’s oral hygiene status had been rated by 298 parents (response rate: 97.4%), and more than a quarter of parents perceived their children to have unfavorable oral hygiene status. HKOHLAT-P were filled by 246 parents (response rate: 80.4%). The average OHL score was 28.70 (±20.14). Parents who refused to fill the HKOHLAT-P questionnaires mainly complained that the questions were too difficult for them to answer. 

### 3.2. Parental Compliance

A total of 253 (82.7%) parents kept their children adhering to the 24-month follow-up study. Compared with children who adhered to follow-up, children who dropped out from the OHE program were more likely to have unfavorable oral hygiene status (41.3% vs. 26.3%, *p* = 0.038). Likewise, parents whose children had been lost to follow-up were more likely to show negative attitudes towards the education topics than those who adhered to follow-up (26.4% vs. 13.8%, *p* = 0.023). Moreover, parents whose children adhered to the follow-up assessments had higher OHL scores (29.99 ± 19.55) than those parents whose children had dropped out from the OHE program (18.68 ± 22.22, *p* = 0.015). Additionally, parental compliance with investigators’ advice was reported by 266 parents (response rate: 86.9%). Of those, 25 (9.4%) parents disclosed that they had never used the OHE materials at home. Parents who perceived that the education materials were not useful for their children were more likely to show noncompliant behaviors towards the OHE intervention (48.0% vs. 19.9%, *p* = 0.001). Additionally, there were no significant differences in parental compliance among parents with various SES (Table 2). 

### 3.3. Findings from the Binary Logistic Regression Model

When parents’ OHL scores, perception of children’s oral hygiene status, attitudes towards OHE topic, usefulness of the OHE materials, type of OHE materials, SES, and children’s SHCN conditions were considered, the final model of binary logistic regression indicated that self-reported noncompliance was more likely to exist among parents who perceived that the OHE materials were not useful (OR = 3.63, 95% CI 1.56 to 8.47, *p* = 0.003). Parents who showed negative attitudes towards the OHE topics were more likely to let their children drop out from the OHE program (OR = 3.45, 95% CI 1.33 to 8.95, *p* = 0.011). Children whose parents’ OHL scores ranged between 26 to 45 were less likely to drop out from the OHE program than children whose parents had lower OHL scores (OR = 0.17, 95% CI 0.05 to 0.64, *p* = 0.008). Besides, comparing to parents of children with autism, parents whose children had been diagnosed with a developmental delay were more likely to drop out from the OHE program (OR = 5.45, 95% CI 1.59 to 18.74, *p* = 0.007, Table 3). 

## 4. Discussion

This study aimed to investigate parental compliance towards OHE activities for preschool children with SHCN. The main findings implied that parental compliance could be affected by parents’ OHL skills, attitudes towards the OHE topics, perceptions of usefulness of the health education materials, as well as their children’s developmental conditions.

One strength of this study was investigating the relationship between parental compliance and parents’ OHL skills. Health literacy refers to “the personal, cognitive, and social skills which determine the ability of individuals to gain access to, understand, and use information to promote and maintain good health” [7]. Although the importance of health literacy in the general education programs were identified by numbers of studies, the impact of OHL on OHE intervention had been rarely investigated probably because limited reliable tools had been established to assess the functional and conceptual OHL [19]. To the best of our knowledge, this is the first investigation which explored the relationship between OHL and parental compliance towards the OHE intervention. In this study, parents’ OHL was assessed by HKOHLAT-P, which was a validated and reliable instrument for examining Chinese OHL in Pediatric Dentistry [18]. Lower OHL scores existed among parents whose children had dropped out from the OHE program (*p* = 0.015). This was further supported by the binary logistic regression, showing that parents’ OHL skills could affect parents’ compliance towards the scheduled follow-up visits (*p* = 0.030). Other scholars also elucidated that poor health literacy could be a barrier to health education, as individuals who had limited skills in reading the education materials might have less developed skills to act upon the target information [7,20]. Moreover, participants with low OHL skills were reticent in disclosing their reading difficulties in a regular consultation [21]. In further studies, additional supports, such as personal forms of communication, educational outreach services, “teach-back” techniques, or individually modified education materials and decision-aids should be provided to parents who have certain limitations in processing the information presented in the education materials [7,20]. Thus, parents who had limited health literacy could be assisted to get a better understanding of the delivered message.

Another factor associated with parental compliance was parents’ attitudes towards the OHE topics. Existing evidence supported that dental attendance, toothbrushing, and snacking habits were significantly associated with children’s oral health status [22,23]. Therefore, the topics delivered in this education program included toothbrushing, tooth-friendly diet, and preparing children for dental visits in their early childhood. Sixteen percent of parents had showed negative attitudes towards at least one of the involved topics, and those parents were more likely to discontinue the OHE intervention for their children (*p* = 0.011). Moreover, parents who perceived that the education materials were not useful were 2.63 times more likely to show noncompliant behaviors towards the OHE intervention (OR = 3.63, 95% CI 1.56 to 8.47, *p* = 0.003). A similar finding was stated in a peer study, in which the authors claimed that high compliance might be positively influenced by the acceptance of the education content [8]. 

Children’s developmental conditions were also related to parental compliance. Parents who were taking care of children with autism showed higher adherence to the follow-up studies. We could not compare this finding with prior studies, as few OHE programs had been conducted among children with SHCN. However, it was reported that visual supports, such as social stories, road maps, visual organizers, visual pictures or even recipes, could make abstract concepts more concrete, and enable autistic children to engage in target messages [24]. Children with autism were able to learn literacy and practice the expected behaviors by using the visual supports [25]. The OHE materials used in this study could be served as visual supports. Parents of autistic children might be more aware of the importance of visual supports than other parents. Thus, those parents were likely to show better compliance towards the OHE intervention.

In the present study, there were no differences in compliance across participants with various socioeconomic backgrounds. This was consistent with a meta-analysis conducted by Coenen and colleagues [26]. In the bivariate analysis, participant adherence to follow-ups was related to parents’ perception of children’s oral hygiene status, parents’ attitudes towards the education topics, and parents’ OHL scores, whereas parental compliance with investigators’ advice was only related to parents’ perception of the usefulness of the OHE material. Since this was a cross-sectional study, the causal effect could not be estimated. However, our findings indicated that, in further studies, efforts should be made to raise parents’ awareness of the importance of OHE, so that the instructions in health education programs would be followed by parents.

The children’s self-reports, as well as fathers’ and mothers’ proxy-reports were the common sources for obtaining children-related information when conducting questionnaire-based studies, whereas the perceptions might be different among children, fathers and mothers [27,28]. Thus, one limitation of this study was that parental compliance towards the investigators’ advice was reported by parents, and we did not distinguish whether those questionnaires were answered by mothers or fathers. This might lead to a potential risk of reporting bias. However, preschool children with SHCN had limited literacy skills to answer the questionnaires, thus the parent-report approach was selected in this study. Additionally, we instructed that the questionnaires should be completed by the parent who had spent more time in taking daily care of the child. As the education topics were embedded with home settings, our initial intention was to investigate the implementation of OHE intervention in children’s daily lives. The main caregivers could provide a representative answer. 

In this study, parents were invited to answer the question “Has your child used the health education materials after you received the materials (Yes/No)”. Parents who responded ‘No’ were indicated as noncompliant with investigators’ advice. Pathirathna and colleagues also used a similar approach to investigate maternal compliance with doctors’ advice [29], whereas Obirikorang et al. adopted a structured questionnaire to investigate patient treatment compliance, which comprised both medication regimen compliance (eight items) and lifestyle modification (5 items) [30]. Apart from the parent-report compliance with the investigators’ advice, we also analyzed participants’ adherence to follow-up studies. Coenen and colleagues also determined compliance by assessing the participant adherence to health promotion programs [26]. By assessing participant adherence to follow-ups and parental compliance with investigators’ advice, an analysis of parental compliance towards the OHE program was provided in this study. However, these crucial variables were not free from the risk of bias because the questions used to collect information related to parental compliance had not been validated. This is another limitation of our study.

The principal findings of this investigation suggested parental noncompliance towards healthcare providers’ advice was more likely to be observed among parents who perceived that the education materials were not useful for their children. A higher dropout rate existed among parents who had limited OHL, or parents who showed negative attitudes towards the health education topics. Children’s developmental conditions were also associated with their adherence to follow-up studies. In future studies, healthcare providers are encouraged to screen the participants whose parents have low health literacy or negative attitudes towards the education content. Moreover, a health education program should be individually tailored to support children whose parents are at higher risks of noncompliant behaviors.

## Figures and Tables

**Table 1:** Participant characteristics.

Variables	*n* (%)	Mean (SD)
Socio-demographic status (*N* = 306)		
Both parents work full time		
Yes	107 (35.0)	
No	199 (65.0)	
Family household income (per month)		
HKD 20,000 or below	121 (39.5)	
Above HKD 20,000	185 (60.5)	
Father’s education level		
9 years or below	61 (19.9)	
10 to 13 years	92 (30.1)	
14 years or above	153 (50.0)	
Mother’s education level		
9 years or below	83 (27.1)	
10 to 13 years	100 (32.7)	
14 years or above	123 (40.2)	
Children’s oral hygiene rated by parents (*N* = 297)		
Favorable	212 (71.4)	
Fair or poor	85 (28.6)	
Negative attitude towards OHE topics (*N* = 306)	49 (16.0)	
OHE material was not useful (*N* = 301)	68 (22.5)	
OHL scores (*N* = 246)		28.70 (20.14)

HKD: Hong Kong Dollars; OHE: Oral Health Education; OHL: Oral Health Literacy; SD: Standard deviation.

**Table 2:** Parental compliance among parents with various OHL, attitudes, perception of children’ oral hygiene status, and SES.

Variables	Adherence to Follow-Ups			Compliance with Investigators’ Advice		
	Yes	No	*p*-Value	Yes	No	*p*-Value
Children’s oral hygiene status			0.038			N.S.
Favorable	185 (73.7)	27 (58.7)		176 (74.3)	17 (68.0)	
Fair or poor	66 (26.3)	19 (41.3)		61 (25.7)	8 (32.0)	
Negative attitude towards OHE	35 (13.8)	14 (26.4)	0.023	41 (17.0)	3 (12.0)	N.S.
OHE material was not useful	62 (24.7)	6 (12.0)	0.05	48 (19.9)	12 (48.0)	0.001
OHL scores	29.99 (19.55)	18.68 (22.22)	0.015	29.15 (19.77)	36.14 (18.50)	N.S.
Parents work full time	87 (34.4)	20 (37.7)	N.S.	76 (31.5)	9 (36.0)	N.S.
Monthly income over HKD 20,000	155 (61.3)	30 (56.6)	N.S.	144 (59.8)	97 (40.2)	N.S.
Father’s education level			N.S.			N.S.
≤9 years	56 (22.1)	5 (9.4)		53 (22.0)	5 (20.0)	
10 to 13 years	74 (29.2)	18 (34.0)		72 (29.9)	4 (16.0)	
≥14 years	123 (48.6)	30 (56.6)		116 (48.1)	16 (64.0)	
Mother’s education level			N.S.			N.S.
≤9 years	74 (29.2)	9 (17.0)		67 (27.8)	6 (24.0)	
10 to 13 years	81 (32.0)	19 (35.8)		79 (32.8)	6 (24.0)	
≥14 years	98 (38.7)	25 (47.2)		95 (39.4)	13 (52.0)	

HKD: Hong Kong Dollars; N.S.: No significance; OHL scores were presented in the format of mean (SD), and the other variables were presented in the format of *n* (%).

**Table 3:** Factors associated with parental compliance during the 24-month follow-up period (final model of binary logistic regression).

Variables	Loss to Follow-Up			Noncompliance with Advice		
	OR	95% CI	*p*-Value	OR	95% CI	*p*-Value
Children’s SHCN conditions						
Autism *			0.048			
Developmental delay	5.45	1.59, 18.74	0.007			
Multiple disabilities	2.28	0.46, 11.24	N.S.			
Other conditions	2.23	0.62, 8.01	N.S.			
Negative attitude towards OHE						
Yes vs. No *	3.45	1.33, 8.95	0.011			
OHE material was not useful						
Not useful vs. useful *				3.63	1.56, 8.47	0.003
OHL scores						
0–25 *			0.03			
26–45	0.17	0.05, 0.64	0.008			
46–52	0.76	0.29, 2.03	N.S.			

* N.S.: No significance; Reference group; SHCN: Special Health Care Need.

## Data Availability

The data set are available from the corresponding author upon reasonable request.
